# Hepatomegaly and Deranged Liver Enzymes in a Patient With Poorly Controlled Type 1 Diabetes Mellitus

**DOI:** 10.1210/jcemcr/luaf100

**Published:** 2025-05-14

**Authors:** George I Habeos, Dimitris Ziazias, Christina Petropoulou, Georgios Eleftherakis, Georgios K Markantes

**Affiliations:** Division of Endocrinology—Department of Internal Medicine, University of Patras School of Health Sciences, Patras PC 26500, Greece; Department of Internal Medicine, University of Patras School of Health Sciences, Patras PC 26500, Greece; Department of Internal Medicine, University of Patras School of Health Sciences, Patras PC 26500, Greece; Department of Internal Medicine, University of Patras School of Health Sciences, Patras PC 26500, Greece; Division of Endocrinology—Department of Internal Medicine, University of Patras School of Health Sciences, Patras PC 26500, Greece

**Keywords:** glycogenic hepatopathy, diabetes mellitus, hepatomegaly, liver function tests, transaminasemia

## Abstract

Glycogenic hepatopathy (GH) is a rare clinical entity characterized by glycogen accumulation in the liver which affects a minority of patients with poorly controlled diabetes mellitus. Its cardinal manifestations include hepatomegaly and elevated liver enzymes. Reaching the diagnosis requires an extensive workup, and typically a liver biopsy. GH is completely reversible with the restoration of good glycemic control. Herein, we report the case of a 20-year-old woman with type 1 diabetes and poor glycemic control, who presented hepatomegaly and a steep increase in transaminase levels while she was being treated for diabetic ketoacidosis. The patient was submitted to a comprehensive laboratory and imaging workup to rule out other possible causes for her deranged liver function and, finally, to a liver biopsy that confirmed the diagnosis of GH. Following the appropriate modifications to her insulin regimen, her glycemic control markedly improved, as did her liver function tests on follow-up.

## Introduction

Elevation of liver enzymes is not unusual in diabetes, and steatotic liver disease (MASLD) is the commonest cause. Although glycogenic hepatopathy (GH) is considered a rare entity, it is a frequent cause of hepatic dysfunction in patients with poorly controlled diabetes, mainly type 1 diabetes mellitus (T1DM). The presented case highlights the challenges of diagnosing GH and advocates for a low threshold of suspicion among clinicians for this serious but reversible disease.

## Case Presentation

A 20-year-old woman was referred to our tertiary center from a district hospital. She had a 3-day history of nausea, lightheadedness, and malaise, without fever, vomiting, diarrhea, or other symptoms. She had a history of T1DM diagnosed at the age of 13, managed with basal-bolus insulin (degludec once daily and lispro with meals). She did not monitor glucose regularly, frequently omitted insulin doses, and administered mealtime insulin empirically, without carbohydrate calculations. Her glycemic control was poor, with frequent severe hyperglycemia or symptomatic hypoglycemia; her last known glycated hemoglobin (HbA1c) was 10% (normal <5.7%), and she had 1 or 2 annual hospitalizations for diabetic ketoacidosis (DKA). She took no other prescribed or over-the-counter medications or supplements, smoked 10 cigarettes daily, and drank alcohol occasionally (<3 units/week). Her father had coronary artery disease; her mother and 2 siblings were healthy.

On the initial examination, she had mild tachypnea (25 breaths/min), dry mucous membranes, and palpable hepatomegaly (3-4 cm below the right costal margin). Her pulse was 110 beats per minute, and blood pressure 110/60 mmHg. Laboratory testing revealed a high anion gap metabolic acidosis (pH 7.07 [normal range 7.35-7.45], PO_2_ 145 mmHg [normal range 75-100 mmHg], PCO_2_ 10.1 mmHg [normal range 35-45 mmHg], HCO_3_ 3 mmol/L [normal range 22-28 mmol/L], AG 29 mmol/L [normal range 4-12 mmol/L]), meeting DKA criteria (glucose 412 mg/dL or 22.9 mmol/L − normal range 70-100 mg/dL or 3.9-5.6 mmol/L, urine ketones 3+). She received aggressive intravenous fluids and insulin. Chest x-ray, electrocardiogram, COVID-19 test, blood/urine cultures, and pregnancy test were negative. She had mild normocytic anemia, marginally elevated lymphocytes, and abnormal liver function tests (LFTs) with predominant aspartate aminotransferase (AST) elevation (Table 1).

**Table 1.** Laboratory tests of the patient during her initial admission and on arrival at our hospital

	Normal range	Initial hospital presentation	Third day of initial admission	Presentation to our hospital
Hemoglobin	12-15.7 g/dL (120-157 g/L)	11.8 g/dL (118 g/L)	11.3 g/dL (113 g/L)	11.1 g/dL (111 g/L)
Mean corpuscular volume	79.0-98.0 fl	90.8 fl	92.1 fl	91.3 fl
White blood cells	4500-10500/μL	4200/μL	4500/μL	4540/μL
Neutrophils	40-75%	41.1%	30.6%	25.6%
Lymphocytes	20-45%	49.5%	58.8%	61.6%
Monocytes	2-10%	6.4%	7.1%	9.2%
Eosinophils	0.5-5%	2.1%	2.4%	2.9%
Basophils	0.3-1%	0.9%	1.1%	0.7%
Platelets	150000-440000/μL	298 000/μL	290 000/μL	275 000/μL
Glucose	70-100 mg/dL (3.9-5.6 mmol/L) (fasting)	412 mg/dL (22.9 mmol/L)	310 mg/dL (17.2 mmol/L)	94 mg/dL (5.2 mmol/L)
Urea	15-54 mg/dL (2.50-8.99 mmol/L)	55 mg/dL (9.16 mmol/L)	19 mg/dL (3.16 mmol/L)	19 mg/dL (3.16 mmol/L)
Creatinine	0.9-1.6 mg/dL (79.6-141.4 μmol/L)	0.5 mg/dL (44.2 μmol/L)	0.5 mg/dL (44.2 μmol/L)	0.6 mg/dL (53.0 μmol/L)
Sodium	134-152 mEq/L (134-152 mmol/L)	138 mEq/L (138 mmol/L)	136 mEq/L (136 mmol/L)	137 mEq/L (137 mmol/L)
Potassium	3.8-5.5 mEq/L (3.8-5.5 mmol/L)	4.2 mEq/L (4.2 mmol/L)	4.5 mEq/L (4.5 mmol/L)	4.7 mEq/L (4.7 mmol/L)
AST	5-40 U/L (0.083-0.667 μkat/L)	149 U/L (2.483 μkat/L)	1408 U/L (23.467 μkat/L)	1184 U/L (19.733 μkat/L)
ALT	5-40 U/L (0.083-0.667 μkat/L)	73 U/L (1.217 μkat/L)	360 U/L (6.0 μkat/L)	357 U/L (5.95 μkat/L)
gGT	10-50 IU/L (0.167-0.833 μkat/L)	63 IU/L (1.05 μkat/L)	88 IU/L (1.467 μkat/L)	116 IU/L (1.933 μkat/L)
ALP	34-104 IU/L (0.567-1.733 μkat/L)	85 IU/L (1.417 μkat/L)	113 IU/L (1.883 μkat/L)	125 IU/L (2.083 μkat/L)
Bilirubin	0.1-1.3 mg/dL (1.71-22.24 μmol/L)	0.20 mg/dL (3.42 μmol/L)	0.20 mg/dL (3.42 μmol/L)	0.38 mg/dL (6.5 μmol/L)
Albumin	3.5-5.5 g/dL (0.53-0.83 mmol/L)	4.0 g/dL (0.60 mmol/L)	3.9 g/dL (0.59 mmol/L)	4.1 g/dL (0.62 mmol/L)
INR	<1.1	1.1	1.0	0.9
C-reactive protein	<0.5 mg/dL (<4.76 nmol/L)	0.40 mg/dL (3.81 nmol/L)	0.45 mg/dL (4.29 nmol/L)	0.22 mg/dL (2.10 nmol/L)
Troponin I	0-15.6 pg/mL (0-15.6 ng/L)	3.1 pg/mL (3.1 ng/L)		0.8 pg/mL (0.8 ng/L)
Creatine kinase	20-140 U/L(0.333-2.333 μkat/L)	24 U/L (0.4 μkat/L)	25 U/L (0.417 μkat/L)	26 U/L (0.433 μkat/L)
Lactate dehydrogenase	120-230 U/L (2-3.833 μkat/L)	636 U/L (10.6 μkat/L)	770 U/L (12.833 μkat/L)	463 U/L (7.717 μkat/L)
Amylase	10-220 U/L (0.167-3.667 μkat/L)	60 U/L (1.0 μkat/L)		85 U/L (1.417 μkat/L)

Values in parentheses are in Système International (SI) units. Abbreviations: ALP, alkaline phosphatase; ALT, alanine transaminase; AST, aspartate transaminase; gGT, gamma-glutamyl transferase; INR, international normalized ratio.

After 24 hours, DKA had resolved, allowing oral intake and transition back to her pre-admission insulin regimen, although glucose levels remained erratic. On day 3, her liver enzymes rose significantly (Table 1), prompting transfer to our hospital for investigation of her hepatomegaly and transaminasemia.

## Diagnostic Assessment

On admission to our hospital, the patient was afebrile, hemodynamically stable, and her initial symptoms had resolved. Clinical examination revealed only hepatomegaly and vulvar redness with white-colored vaginal discharge; there was no splenomegaly, icterus, rash, ascites, or signs of portal hypertension. Arterial blood gases were normal. Laboratory testing showed mild normocytic anemia, relative neutropenia, lymphocytosis, and elevated LFTs (Table 1).

The patient admitted discontinuing long-acting insulin 2 to 3 years ago, relying solely on rapid-acting insulin in high doses to correct hyperglycemia. She reported daily hypoglycemia, countered by excessive carbohydrate intake.

Abdominal ultrasound showed a diffusely enlarged, hyperechogenic liver with otherwise normal intra-abdominal organs and no ascites or hepatic vein thrombosis. Computed tomography (CT) confirmed hepatomegaly and the absence of structural abnormalities, with the liver and spleen displaying similar density. Further tests were conducted to identify potential causes of hepatomegaly and liver dysfunction (Table 2).

**Table 2.** Laboratory evaluation of the patient's abnormal liver biochemistry

Parameter	Reference range	Patient's value
Hepatitis A IgM antibody	NEGATIVE	NEGATIVE
Hepatitis A IgG antibody	NEGATIVE	POSITIVE
HBsAg	NEGATIVE	NEGATIVE
Anti-HBs	NEGATIVE	NEGATIVE
Anti-HBc IgG	NEGATIVE	POSITIVE
Anti-HBc IgM	NEGATIVE	NEGATIVE
HBeAg	NEGATIVE	NEGATIVE
Anti-HBe	NEGATIVE	NEGATIVE
HBV DNA	NEGATIVE	NEGATIVE
Hepatitis C antibodies	NEGATIVE	NEGATIVE
HIV antibodies	NEGATIVE	NEGATIVE
Rapid plasma regain test (RPR)	NEGATIVE	NEGATIVE
Cytomegalovirus IgM antibody	NEGATIVE	NEGATIVE
Cytomegalovirus IgG antibody	NEGATIVE	POSITIVE
Epstein-Barr virus IgM antibody	NEGATIVE	NEGATIVE
Epstein-Barr virus IgG antibody	NEGATIVE	POSITIVE
Toxoplasma IgM antibody	NEGATIVE	NEGATIVE
Toxoplasma IgG antibody	NEGATIVE	NEGATIVE
Leptospira antibodies	NEGATIVE	NEGATIVE
Leishmania antibodies	NEGATIVE	NEGATIVE
Antimitochondrial antibody	NEGATIVE	NEGATIVE
Anti-smooth muscle antibody	NEGATIVE	NEGATIVE
Anti–liver-kidney microsomal antibody	NEGATIVE	NEGATIVE
Rheumatoid factor	NEGATIVE	NEGATIVE
Anti-nuclear antibody	NEGATIVE	NEGATIVE
Anti-centromere antibody	NEGATIVE	NEGATIVE
Antibody to ribonucleoprotein	NEGATIVE	NEGATIVE
Anti-topoisomerase antibody	NEGATIVE	NEGATIVE
Anti-neutrophil cytoplasmic antibodies (cANCA and pANCA)	NEGATIVE	NEGATIVE
Tissue transglutaminase antibodies	NEGATIVE	NEGATIVE
C3	79-152 mg/dL (0.79-1.52 g/L)	120 mg/dL (1.2 g/L)
C4	16-38 mg/dL (0.16-0.38 g/L)	25 mg/dL (0.25 g/L)
Serum immunoglobulin electrophoresis	IgA: 82-453 mg/dL (0.82-4.53 g/L) IgG: 751-1560 mg/dL (7.51-15.6 g/L) IgM: 46-304 mg/dL (0.46-3.04 g/L) IgE: 5-165 IU/mL (0.012-0.396 g/L)	IgA: 639 mg/dL (6.39 g/L) IgG: 1280 mg/dL (12.8 g/L) IgM: 91 mg/dL (0.91 g/L) IgE: 223 IU/mL (0.535 g/L)
Ceruloplasmin	22-58 mg/dL (1.64-4.33 μmol/L)	47 mg/dL (3.51 μmol/L)
Alpha-1 antitrypsin	92-224 mg/dL (16.93-41.21 μmol/L)	165 mg/dL (30.36 μmol/L)
Serum iron	59-158 μg/dL (10.57-28.29 μmol/L)	51 μg/dL (9.13 μmol/L)
Total iron binding capacity	149-492 μg/dL (26.68-88.10 μmol/L)	465 μg/dL (83.27 μmol/L)
Ferritin	6.9-283 ng/mL (0.02-0.64 nmol/L)	70 ng/mL (0.16 nmol/L)
Alpha fetoprotein	0-15 ng/mL (0-15 μg/L)	2.05 ng/mL (2.05 μg/L)
Peripheral blood smear		Mildly elevated lymphocyte number—increased activated lymphocytes, all other cell types normal
Flow cytometry		Numbers and proportions of blood lymphocyte subsets normal, no abnormal cells detected

Values in parentheses are in Système International (SI) units. Abbreviations: Anti-HBc, antibody against hepatitis B core antigen; Anti-HBe, antibody against hepatitis B e antigen; Anti-HBs, antibody against Australia antigen; C3, complement component 3; C4, complement component 4; HBeAg, hepatitis B e antigen; HBsAg, Australia antigen; HBV, hepatitis B virus; HIV, human immunodeficiency virus.

Endocrine workup showed elevated HbA1c (9.6%) and undetectable C-peptide. Serum thyrotropin (TSH) was mildly elevated (4.7 mIU/L [normal range 0.27-4.50 mIU/L]), with normal free thyroxin and negative anti-thyroid peroxidase antibodies.

Ophthalmologic examination revealed right-eye cataract but no diabetic retinopathy. Vaginal swab microscopy showed clue cells, and cultures detected Ureaplasma and Candida. The rest of the gynecological examination was unremarkable, with transvaginal ultrasound confirming a normal uterus and ovaries.

## Treatment

**Figure 1.**
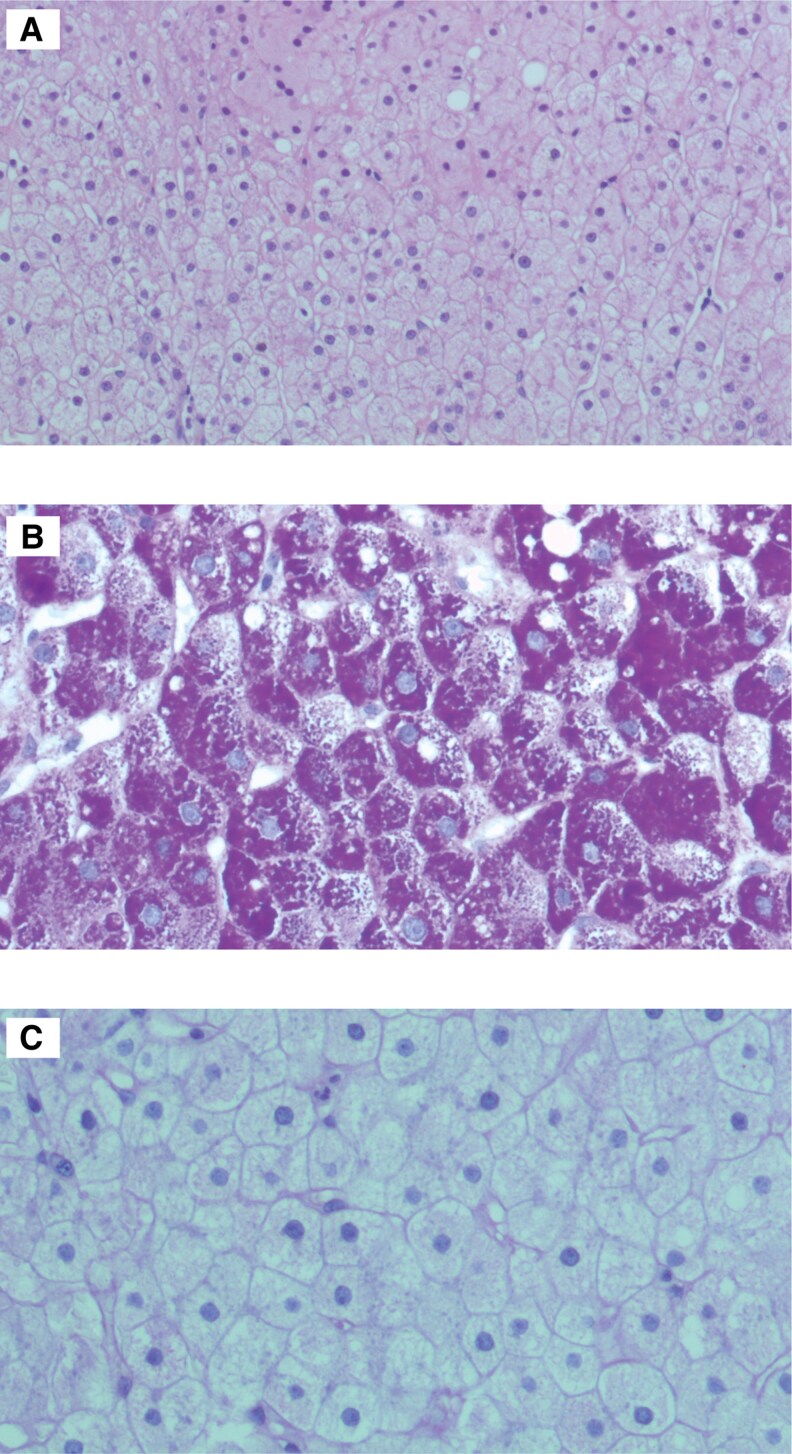
Histologic appearance of the patient's liver biopsy sample. (A) hematoxylin and eosin (B) periodic acid–Schiff (PAS) (C) PAS-diastase.

The patient was started on fluconazole, metronidazole, and doxycycline for the mixed bacterial/fungal genital infection. Her long-acting insulin dose was reduced due to frequent fasting hypoglycemia, and she received intensive training on meal carbohydrate calculation. Given her clinical profile and the lack of alternative explanations for hepatomegaly and transaminasemia, GH was strongly suspected, prompting a liver biopsy. Histology revealed mildly enlarged, pale hepatocytes with increased cytoplasmic volume and glycogenated nuclei (hematoxylin and eosin stain) (Fig. 1A). Periodic acid–Schiff (PAS) stain showed strong magenta hepatocyte staining (Fig. 1B), which disappeared with diastase treatment, forming “ghost cells” (Fig. 1C). Mild steatosis was present in 5% to 10% of hepatocytes, but no inflammation, fibrosis, or necrosis was observed.

## Outcome and Follow-Up

The course of the patient's LFTs and glucose levels is shown in Table 3. Peak transaminase levels occurred on day 2 of admission but declined rapidly with improved glycemic control. Alkaline phosphatase (ALP) was only mildly elevated, while bilirubin, albumin, and international normalized ratio (INR) remained normal. At a one-month follow-up, AST, alanine aminotransferase (ALT), and gamma-glutamyl transferase (gGT) had further decreased but remained abnormal. Hepatomegaly persisted (1-2 cm below the right costal margin), albeit reduced, and the patient reported significantly improved glycemic control.

**Table 3.** Liver function and glucose tests of the patient during her admission in our hospital and on follow-up visit 1 month after discharge from the hospital

	Normal range	Admission	Day 2	Day 4	Day 6	Discharge	1 month post-discharge
AST	5-40 U/L (0.083-0.667 μkat/L)	1184 U/L (19.733 μkat/L)	1713 U/L (28.55 μkat/L)	476 U/L (7.933 μkat/L)	124 U/L (2.067 μkat/L)	139 U/L (2.317 μkat/L)	85 U/L (1.417 μkat/L)
ALT	5-40 U/L (0.083-0.667 μkat/L)	357 U/L (5.95 μkat/L)	646 U/L (10.767 μkat/L)	457 U/L (7.617 μkat/L)	198 U/L (3.3 μkat/L)	186 U/L (3.1 μkat/L)	138 U/L (2.3 μkat/L)
gGT	10-50 IU/L (0.167-0.833 μkat/L)	116 IU/L (1.933 μkat/L)	127 IU/L (2.117 μkat/L)	169 IU/L (2.817 μkat/L)	155 IU/L (2.583 μkat/L)	137 IU/L (2.283 μkat/L)	63 IU/L (1.05 μkat/L)
ALP	34-104 IU/L (0.567-1.733 μkat/L)	125 IU/L (2.083 μkat/L)	118 IU/L (1.967 μkat/L)	160 IU/L (2.667 μkat/L)	120 IU/L (2.0 μkat/L)	107 IU/L (1.783 μkat/L)	98 IU/L (1.633 μkat/L)
Bilirubin	0.1-1.3 mg/dL (1.71-22.24 μmol/L)	0.38 mg/dL (6.50 μmol/L)	0.30 mg/dL (5.13 μmol/L)	0.32 mg/dL (5.47 μmol/L)	0.30 mg/dL (5.13 μmol/L)	0.23 mg/dL (3.93 μmol/L)	0.43 mg/dL (7.36 μmol/L)
Albumin	3.5-5.5 g/dL (0.53-0.83 mmol/L)	4.10 g/dL (0.62 mmol/L)	3.90 g/dL (0.59 mmol/L)	4.60 g/dL (0.69 mmol/L)	4.10 g/dL (0.62 mmol/L)	4.00 g/dL (0.60 mmol/L)	3.80 g/dL (0.57 mmol/L)
INR	<1.1	0.90	1.00		0.92	1.10	1.00
Glucose fasting/random	70-100 mg/dL (3.9-5.6 mmol/L) (fasting)	94 mg/dL (5.2 mmol/L)/313 mg/dL (17.4 mmol/L), 114 mg/dL (6.3 mmol/L)	58 mg/dL (3.2 mmol/L)/287 mg/dL (15.9 mmol/L), 219 mg/dL (12.2 mmol/L)	73 mg/dL (4.1 mmol/L)/232 mg/dL (12.9 mmol/L), 147 mg/dL (8.2 mmol/L)	128 mg/dL (7.1 mmol/L)/209 mg/dL (11.6 mmol/L), 156 mg/dL (8.7 mmol/L)	97 mg/dL (5.4 mmol/L)/135 mg/dL (7.5 mmol/L)	−/141 mg/dL (7.8 mmol/L)

Values in parentheses are in Système International (SI) units. Abbreviations: ALP, alkaline phosphatase; ALT, alanine transaminase; AST, aspartate transaminase; gGT, gamma-glutamyl transferase; INR, international normalized ratio.

## Discussion

GH is a rare complication of diabetes, manifesting with hepatomegaly and elevated liver enzymes. It is primarily seen in autoimmune diabetes—T1DM with poor glycemic control but has also been reported in patients with type 2 diabetes (T2DM) [1, 2]. The pathogenesis involves excess glycogen accumulation in hepatocytes, a reversible process with improved glycemic control [1, 2].

The true incidence of GH is unknown. With approximately 150 reported cases in the literature [2], it is generally considered a rare clinical condition. However, it is possible that it is underdiagnosed, due to decreased clinician awareness and inability of the ultrasound to distinguish it from the much commoner MASLD [3-5].

**Figure 2.**
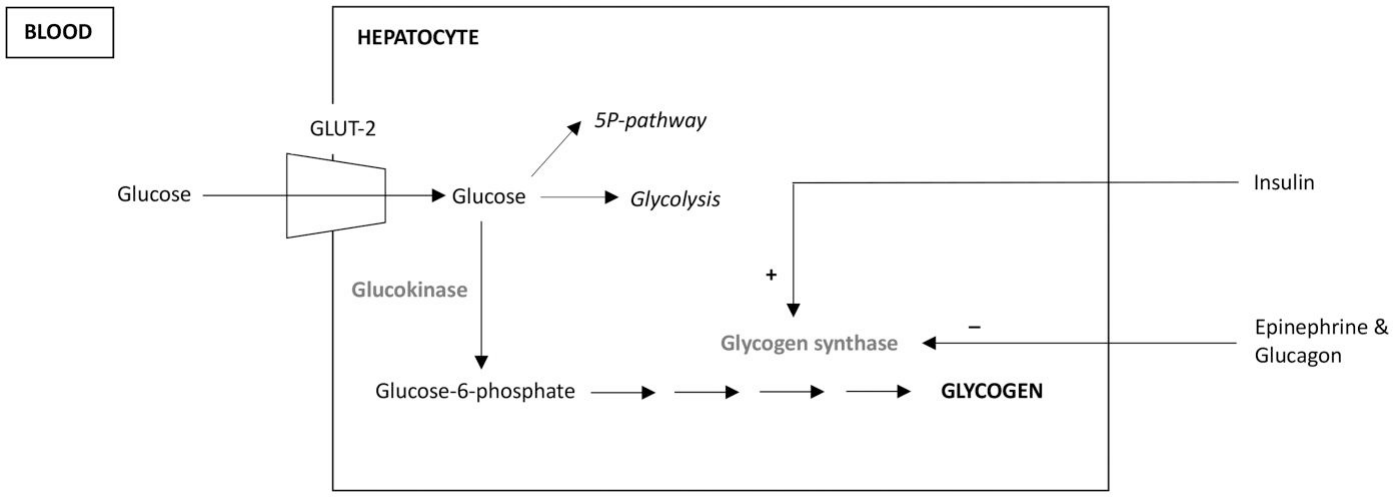
Physiology of hepatic glycogen accumulation in glycogenic hepatopathy. Glucose enters the hepatocyte independently of insulin, and it is phosphorylated to glucose-6-phosphate by glucokinase, an irreversible reaction that traps glucose in the cell. Glucose-6-phosphate can follow the glycolytic pathway (oxidation), the pentose phosphate pathway (lipid and nucleic acid synthesis), or be stored in the form of glycogen. Which of these processes prevails at a given time depends on the balance between the available and required energy in the cell, as well as on the hormonal milieu: insulin promotes glycolysis and glycogen synthesis (by activating glycogen synthase and inhibiting glycogen phosphorylase), while epinephrine and glucagon promote glycogen breakdown. Abbreviations: 5P-pathway, pentose phosphate pathway; GLUT-2, glucose transporter 2.

Intracellular glycogen accumulation is the hallmark of GH. The fate of glucose in the hepatocyte is shown in Fig. 2. For GH to occur, there are 2 prerequisites: the person with diabetes must present marked or prolonged hyperglycemia and be administered high insulin doses [6], explaining why GH prevalence was higher before the advent of long-acting insulin analogs, when patients were treated with high doses of short-acting insulin. Our patient was a strong candidate for GH, given her marked hyperglycemia and overzealous, exclusive use of short-acting insulin.

Why GH affects only a minority of T1DM patients remains unclear. Investigators have suggested defective glycogen synthesis and/or breakdown mechanisms in affected individuals. GH is strongly associated with DKA [2]; a recent case-control study showed that among patients with T1DM, those with GH had worse overall glycemic control and were far more likely to have recurrent episodes of DKA [7]. Case series confirm consistently poor glycemic control in GH patients (HbA1c 11%-11.5%) in pediatric [8] and adult cohorts [1, 7]. DKA is a surrogate of poor glycemic control, but it is also characterized by metabolic alterations potentially promoting glycogen buildup: increased levels of cortisol, adrenaline, and growth hormone stimulate lipolysis and release of free fatty acids from adipose tissue. High circulating levels of free fatty acids may suppress glucose oxidation, promoting its storage as glycogen. Furthermore, the hypophosphatemia usually accompanying DKA, reflecting intracellular phosphate deficit, could limit the enzymatic activity of glycogen phosphorylase kinase, thereby reducing the ability of the hepatocytes to degrade glycogen [9]. Our patient had DKA; her serum phosphate was not measured on initial admission, but it was normal on subsequent measurements.

The clinical picture of GH varies: some patients are completely asymptomatic, others present with mild right upper quadrant or diffuse abdominal pain. Rare cases present as acute hepatitis (pruritus, jaundice) or ascites. Hepatomegaly, seen in >90% of cases, is the cardinal clinical sign. Right upper quadrant tenderness is common, while splenomegaly is typically absent [2]. The main laboratory finding is transaminase elevation (hepatocellular pattern), the magnitude of which varies from mild to more than 100-fold increase over the normal range; markedly increased aminotransferases are usually observed in patients presenting with DKA while in those not experiencing a DKA episode, the derangement is milder [2]. In approximately 60% of the reported cases AST was elevated more than ALT, with pronounced AST predominance (AST/ALT ratio >2) in 25% [1, 2]. ALP, gGT, and bilirubin are usually mildly increased or normal, and the liver synthetic function is typically preserved (normal prothrombin time, INR, and serum albumin levels). The mechanism underpinning liver enzyme elevation in GH is unclear; enzyme leakage due to hepatocyte membrane injury has been hypothesized [10]. The reason why AST elevation is usually more pronounced is also elusive. Our patient had hepatomegaly, mild diffuse abdominal tenderness, significant transaminase and mild gGT and ALP elevations, and normal bilirubin, albumin, and INR. Transaminasemia with AST predominance might also originate from non-hepatic sources, as in muscle damage (eg, rhabdomyolysis, myositis, myocardial necrosis) or hemolysis [11]; the patient's presentation and evaluation (electrocardiogram, creatine kinase, troponin, bilirubin, blood smear) were not compatible with such diagnoses.

The differential diagnosis of hepatomegaly and transaminasemia is broad and includes numerous diseases (Table 4). The most common cause of elevated liver enzymes in the general population as well as in diabetes is MASLD [3-5]. In people with T1DM, the differential diagnosis of deranged LFTs should always include hepatosclerosis, a manifestation of diabetic microangiopathy which is usually associated with severe microangiopathy in other organs [12]. Imaging studies cannot provide a definitive diagnosis in GH. Ultrasound findings are similar to MASLD (hepatomegaly and increased echogenicity) [13]. In CT the liver is bright compared to the spleen, while the opposite happens in MASLD [14]. Gradient-dual-echo magnetic resonance imaging (MRI) can distinguish GH from MASLD: GH shows no significant difference in signal intensity between in phase and opposed-phase T1 weighted gradient-dual-echo MRI images, while MASLD does [15].

**Table 4.** Differential diagnosis of hepatomegaly and elevated transaminases

MASLD
Alcoholic hepatitis
Viral hepatitis
Autoimmune hepatitis
Glycogen storage diseases
Hemochromatosis
Wilson disease
Alpha-1 antitrypsin deficiency
HELLP syndrome
Fatty liver of pregnancy
Medication
Hepatosclerosis
Congestive heart failure
Budd-Chiari syndrome
Infectious diseases
Hematological diseases

Abbreviations: HELLP, hemolysis, elevated liver enzymes, low platelet count; MASLD, metabolic dysfunction-associated steatotic liver disease.

Our patient's acute LFT elevation during a DKA episode could also raise suspicion of ischemic hepatitis due to dehydration-induced hemodynamic compromise. Ischemic hepatitis is typically characterized by a rapid, massive, and transient rise in serum aminotransferase and lactate dehydrogenase (LDH) levels in the context of severe systemic hypotension. ALP and bilirubin are usually mildly elevated, and the liver's synthetic function is normal or mildly impaired [16]. Patients with ischemic hepatitis typically have evidence of other organ hypoperfusion, such as altered mental status or acute kidney injury. The histologic hallmark of ischemic hepatitis is centrilobular necrosis [16]. Though initially dehydrated, our patient remained normotensive throughout her admission, without end-organ damage; her LDH was increased, but not dramatically. Moreover, she did not have any history or signs of cardiac disease or other pathology potentially leading to hepatic congestion; the latter are considered, along with systemic hypotension, as prerequisites for the manifestation of ischemic hepatitis [16]. Overall, our patient's history, clinical presentation, and laboratory/imaging evaluation supported GH, by excluding other possibilities.

Liver biopsy is the gold standard for GH diagnosis. Hallmarks include significant glycogen accumulation in the hepatocytes, which appear swollen, and no or mild steatosis and inflammation; fibrosis is usually absent. On hematoxylin and eosin stain hepatocytes appear pale and distended, with increased cytoplasmic volume, accentuation of cell membranes, and many glycogenated nuclei. Intracellular glycogen deposits can be clearly visualized magenta with PAS stain, which disappears after treatment with PAS-D (hepatocytes turn from purple to pale-“ghost cells”) [1, 2]. Our patient's liver biopsy confirmed glycogen accumulation, while the absence of necrosis excluded ischemic hepatitis (Fig. 1).

GH lacks a specific treatment. Restoration of good glycemic control is the only available management option, resulting in regression of hepatomegaly and normalization of LFTs within days to weeks or even months. GH is considered fully reversible [17]; it may recur if uncontrolled hyperglycemia reappears [18], but it does not generally progress to cirrhosis. Among the many reported cases of GH, there are a few with striking similarities to ours. Cha et al presented 2 young adult females with poorly controlled T1DM and marked, acute aminotransferase elevation, with an AST/ALT ratio of >2; in both, GH was histologically proven and LFTs rapidly improved [19]. Ikarashi et al reported another 2 women with T1DM and multiple episodes of DKA, who presented with AST-predominant transaminasemia. They were diagnosed with GH after biopsy, and their LFTs decreased; both experienced relapses in LFTs derangement, presumably due to sustained poor glycemic control [18]. In our case, improvement of the patient's glycemia led to partial regression of her hepatomegaly and transaminasemia, 4 weeks after her discharge.

## Learning Points

- GH is a rare disease, most commonly affecting patients with poorly controlled T1DM.
- It should always be included in the differential diagnosis of deranged LFTs in people with diabetes, especially if there is concurrent hepatomegaly.
- Diagnosis of GH requires advanced imaging and, typically, liver biopsy.
- Implementation of strict glycemic control leads to complete reversal of the disease.

## Contributors

All authors made individual contributions to authorship. D.Z., C.P., G.E., and G.K.M. were involved in the diagnosis and management of the patient. G.I.H. and G.K.M. were involved in the literature review and the preparation of the original manuscript draft. D.Z., C.P., and G.E. were involved in the original draft editing. All authors reviewed and approved the final draft.

## Data Availability

Original data generated and analyzed during this study are included in this published article.

## Abbreviations

ALP: alkaline phosphatase
ALT: alanine aminotransferase
AST: aspartate aminotransferase
CT: computed tomography
DKA: diabetic ketoacidosis
gGT: gamma-glutamyl transferase
GH: glycogenic hepatopathy
HbA1c: glycated hemoglobin
INR: international normalized ratio
LDH: lactate dehydrogenase
LFT: liver function test
MASLD: metabolic dysfunction–associated steatotic liver disease
PAS: Periodic acid–Schiff (stain)
T1DM: type 1 diabetes mellitus
